# TRAF4 is crucial for ST2^+^ memory Th2 cell expansion in IL-33–driven airway inflammation

**DOI:** 10.1172/jci.insight.169736

**Published:** 2023-09-22

**Authors:** Jianxin Xiao, Xing Chen, Weiwei Liu, Wen Qian, Katarzyna Bulek, Lingzi Hong, William Miller-Little, Xiaoxia Li, Caini Liu

**Affiliations:** 1 Inflammation and Immunity, Cleveland Clinic Lerner Research Institute, Cleveland, Ohio, USA.; 2 Medical Scientist Training Program,; 3 Department of Pathology, and; 4 Department of Molecular Medicine, Case Western Reserve University School of Medicine, Cleveland, Ohio, USA.

**Keywords:** Immunology, Inflammation, Allergy, Cellular immune response, Cytokines

## Abstract

Tumor necrosis factor receptor–associated factor 4 (TRAF4) is an important regulator of type 2 responses in the airway; however, the underlying cellular and molecular mechanisms remain elusive. Herein, we generated T cell–specific TRAF4-deficient (CD4-cre *Traf4^fl/fl^*) mice and investigated the role of TRAF4 in memory Th2 cells expressing IL-33 receptor (ST2, suppression of tumorigenicity 2) (ST2^+^ mTh2 cells) in IL-33–mediated type 2 airway inflammation. We found that in vitro–polarized TRAF4-deficient (CD4-cre *Traf4^fl/fl^*) ST2^+^ mTh2 cells exhibited decreased IL-33–induced proliferation as compared with TRAF4-sufficient (*Traf4^fl/fl^*) cells. Moreover, CD4-cre *Traf4^fl/fl^* mice showed less ST2^+^ mTh2 cell proliferation and eosinophilic infiltration in the lungs than *Traf4^fl/fl^* mice in the preclinical models of IL-33–mediated type 2 airway inflammation. Mechanistically, we discovered that TRAF4 was required for the activation of AKT/mTOR and ERK1/2 signaling pathways as well as the expression of transcription factor *Myc* and nutrient transporters (*Slc2a1*, *Slc7a1*, and *Slc7a5*), signature genes involved in T cell growth and proliferation, in ST2^+^ mTh2 cells stimulated by IL-33. Taken together, the current study reveals a role of TRAF4 in ST2^+^ mTh2 cells in IL-33–mediated type 2 pulmonary inflammation, opening up avenues for the development of new therapeutic strategies.

## Graphical abstract

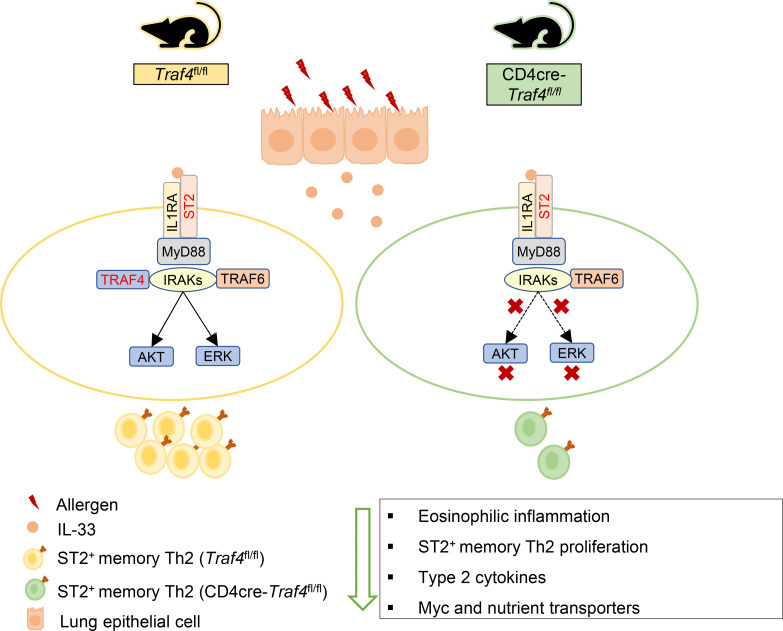

## Introduction

The alarmin cytokine IL-33, produced mainly by structural cells including endothelial, epithelial, and smooth muscle cells, is a well-known inducer and amplifier of type 2 immune responses in airway inflammation (1–5). IL-33 and its receptor subunit ST2 (suppression of tumorigenicity 2, encoded by *IL1RL1*) levels are elevated in asthmatic patients and mice and linked to the severity and steroid resistance of asthma (6–9). Furthermore, polymorphisms in *IL33* and *IL1RL1* have been associated with an increased risk of asthma in several genome-wide association studies involving a diverse range of geographical populations (1–5, 10). In addition, people carrying a loss-of-function mutation of *IL33* have reduced blood eosinophil counts and are protected from asthma (11). Genetic ablation or antibody blockade of the IL-33/ST2 axis ameliorates airway inflammation in multiple preclinical models of allergic asthma (12–14). More interestingly, the results from 2 recent phase II clinical trials show that monoclonal antibodies targeting IL-33 (itepekimab) or ST2 (astegolimab) are beneficial to patients with asthma by reducing blood eosinophils, improving lung function, or lowering exacerbation rate (15, 16).

IL-33 signals through a heterodimeric receptor complex consisting of ST2 and IL-1 receptor accessory protein. The activation of the IL-33 receptor complex leads to the recruitment of myeloid differentiation primary response protein 8 (MYD88), tumor necrosis factor (TNF) receptor‑associated factor 6 (TRAF6), and IL-1 receptor–associated kinases (IRAKs), which results in activation of downstream pathways including mitogen-activated protein kinases (MAPKs), nuclear factor κB (NF-κB), AKT/mTOR, and more (17). IL-33 targets multiple innate and adaptive cell types (e.g., group 2 innate lymphocytes [ILC2s], mast cells, eosinophils, basophils, macrophages, DCs, Th2 cells, and Tregs) that are important players in type 2 immunity (17, 18).

IL-33–responsive ST2^+^ memory Th2 (mTh2) subsets play crucial roles in type 2 allergic airway inflammation. In humans, ST2-expressing mTh2 cells were confined or elevated in individuals with allergic airway diseases (19–21). In mice subjected to a house dust mite–induced (HDM-induced) allergic asthma model, both circulating and resident ST2^+^ mTh2 (Trm-Th2) cells were shown to cooperatively promote the development of full-blown type 2 airway inflammation (22). ST2^+^ Trm-Th2 cells resided in a lymphoid-like structure called inducible bronchus-associated lymphoid tissue formed in the lung during chronic allergic inflammation and were maintained by IL-7– and IL-33– producing lymphatic endothelial cells (23). Direct administration of IL-33 into mouse airways resulted in increased numbers of lung tissue-localized ST2^+^CD44^hi^ mTh2 cells with enhanced production of IL-5 and IL-13 (9). Repetitive exposure of HDM to mice induced a significant increase in collagen deposition and increased numbers of ST2^+^ mTh2 cells producing amphiregulin (encoded by *Areg*, an important growth factor for tissue repair) in lung tissue (8). In an ovalbumin-induced (OVA-induced) relapsing-remitting mouse model of allergic asthma, Th2 cytokine–producing ST2^+^CD44^+^CD69^+^ Trm-Th2 cells were found to mediate allergen-induced disease relapse and maintain “allergic memory” in the lung for the lifetime of the mice (24).

The RING domain E3 ubiquitin ligase TRAF4 belongs to the TRAF family, which consists of 7 members (TRAF1–7) (25–27). It acts either as a negative or positive regulator in multiple cytokine/growth factor signaling pathways (e.g., EGF, TGF-β, NOD2, IL-17 family, etc.), modulating cancer development and inflammatory diseases including asthma (25–28). TRAF4-deficient mice showed attenuated OVA-induced or IL-25–induced (IL-17E) type 2 pulmonary inflammation, implying that TRAF4 is an important regulator in these processes (29, 30). However, the cellular and molecular mechanisms of TRAF4 in type 2 immunity are still unclear.

In the current study, we generated T cell–specific TRAF4-deficient mice to investigate the intrinsic role of TRAF4 in ST2^+^ mTh2 cells in IL-33–mediated type 2 airway inflammation. The results from the present study indicate that TRAF4 is critical for the proliferation of ST2^+^ mTh2 induced by IL-33 in both in vitro and in vivo studies. Mechanistically, we demonstrated that TRAF4 is an essential signaling molecule for IL-33–mediated AKT/mTOR and ERK1/2 pathways, as well as for the expression of signature genes associated with T cell growth and proliferation in ST2^+^ mTh2 cells.

## Results

### TRAF4 deficiency impairs IL-33–mediated proliferation of in vitro–polarized ST2^+^ mTh2 cells.

**Figure 1:**
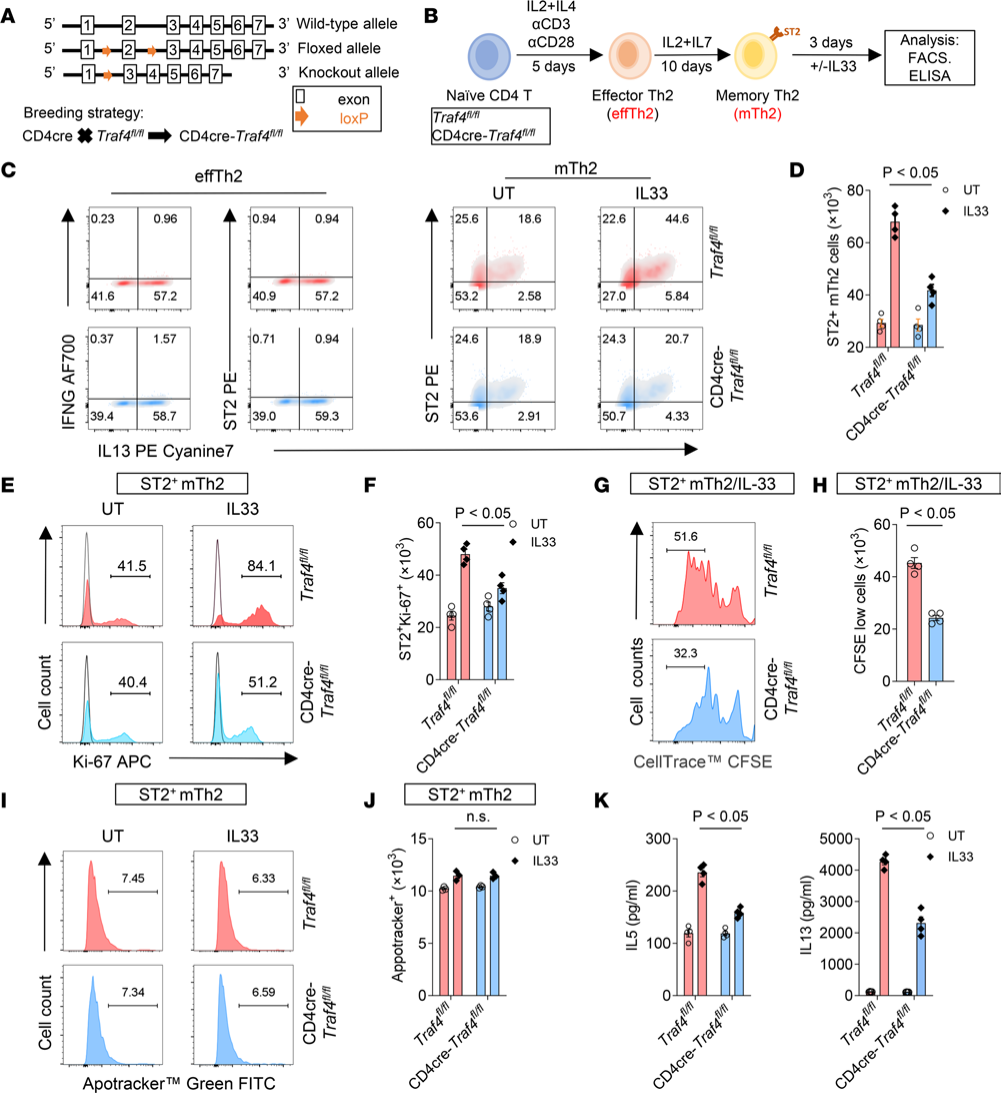
TRAF4 deficiency impairs IL-33–mediated proliferation of in vitro–polarized ST2^+^ mTh2 cells. (**A**) Wild-type, floxed, and conditional knockout alleles of mouse *Traf4* are depicted schematically. (**B**) Naive CD4^+^ T cells, isolated from T cell–specific TRAF4-deficient (CD4-cre *Traf4^fl/fl^*) and TRAF4-sufficient (*Traf4^fl/fl^*) mice, were differentiated under Th2 conditions for 5 days to generate effector Th2 (effTh2) cells; effTh2 cells were then cultured (with IL-2 and IL-7 only) for 10 days to obtain mTh2 cells (mTh2). mTh2 cells were then treated with sham (UT) or IL-33 for 3 days before indicated analysis (**C**–**K**). (**C**) Representative flow cytometry plots of effTh2 and sham- and IL-33–treated mTh2 cells. (**D**) Absolute numbers of ST2^+^ mTh2 cells. (**E**) Histograms of Ki-67^+^ST2^+^ mTh2 cells. Filled histograms represent the cell population stained by the Ki-67 antibody, and the unfilled histograms represent the cell population stained by the isotype control antibody. (**F**) Absolute numbers of Ki-67^+^ST2^+^ mTh2 cells. (**G**) Histograms of IL-33–treated ST2^+^ mTh2 cells subjected to CFSE cell proliferation assay. (**H**) Absolute numbers of CFSE^lo^ cells. (**I**) Histograms of apoptotic mTh2 cells (FITC^+^). (**J**) Absolute numbers of apoptotic mTh2 cells. (**K**) IL-5 and IL-13 protein concentrations in cell medium were quantified by ELISA. Plotted data were shown as means ± SEM. Statistical analysis was performed with 1-way ANOVA (**H**) or 2-way ANOVA (**D**, **F**, **G**, and **K**). All data are representative of 3 independent experiments.

Previous studies showed that TRAF4 is an important regulator of type 2 responses in murine allergic asthma models (29, 30). To explore the precise molecular and cellular mechanisms of TRAF4 in type 2 airway inflammation, we generated *Traf4^fl/fl^* mice with a floxed allele for *Traf4* by genetically targeting exon 2 of *Traf4* (Figure 1A). To investigate the role of TRAF4 in T cells, we bred CD4-cre–transgenic mice onto *Traf4^fl/fl^* mice to generate T cell–specific TRAF4-deficient mice (CD4-cre *Traf4^fl/fl^*). Naive T cells (CD4^+^CD44^–^CD62L^+^) isolated from TRAF4-sufficient (*Traf4^fl/fl^*) mice and TRAF4-deficient (CD4-cre *Traf4^fl/fl^*) were cultured under Th2-polarizing conditions (IL-2, IL-4, anti–IFN-γ Ab) in the presence of TCR stimulation (anti-CD3) and costimulation (anti-CD28) for 5 days to generate ST2^–^ effector Th2 (effTh2) cells. Then effTh2 cells were removed from anti-CD3/anti-CD28 stimulation and cultured with IL-2 and IL-7 for an additional 10 days to generate ST2^+^ mTh2 cells (Figure 1B). We found that both TRAF4-deficient (CD4-cre *Traf4^fl/fl^*) and control (*Traf4^fl/fl^*) cells differentiated into similar percentages and numbers of IL-13^+^ST2^–^ effTh2 cells under IL-4/IL-2–driven Th2-polarizing conditions and IL-13^+^ST2^+^ mTh2 cells under IL-2/IL-7–culturing conditions (Figure 1, C and D). However, after IL-33 treatment of ST2^+^ mTh2 cells for another 3 days, we discovered that a higher percentage and number of ST2^+^ mTh2 cells were induced in *Traf4^fl/fl^* culture than in CD4-cre *Traf4^fl/fl^* culture (Figure 1, C and D). In addition, ST2^+^ cells in IL-33–treated *Traf4^fl/fl^* mTh2 culture displayed greater proliferating potential (as indicated by Ki-67 staining) as compared with ST2^+^ cells in IL-33–treated CD4-cre *Traf4^fl/fl^* mTh2 culture (Figure 1, E and F). Consistently, cell tracing experiments with CFSE also showed upon IL-33 stimulation, ST2^+^ mTh2 cells generated from *Traf4^fl/fl^* mice divided faster than ST2^+^ mTh2 cells from CD4-cre *Traf4^fl/fl^* mice (Figure 1, G and H). In contrast, the apoptosis rate of ST2^+^ cells was similar in TRAF4-sufficient (*Traf4^fl/fl^*) and TRAF4-deficient (CD4-cre *Traf4^fl/fl^*) mTh2 cells (Figure 1, I and J). IL-33–induced production of type 2 cytokines (IL-5 and IL-13) in culture supernatant was also significantly reduced in TRAF4-deficient mTh2 cells as compared with those in control cells (Figure 1K). The above results indicate that TRAF4 is critical for IL-33–induced ST2^+^ mTh2 cell proliferation but dispensable for IL-2/IL-4–driven polarization of effTh2 and IL-2/IL-7–driven development of ST2-expressing mTh2 cells. Prior work has indicated that IL-33 enhances the development of ST2^+^ Tregs (31–33). To investigate the role of IL-33/TRAF4 axis in ST2^+^ Treg development, we polarized naive CD4^+^ T cells from TRAF4-deficient (CD4-cre *Traf4^fl/fl^*) and TRAF4-sufficient (*Traf4^fl/fl^*) mice under the Treg condition in the presence of IL-33. Our results, presented in Supplemental Figure 2, A and B (supplemental material available online with this article; https://doi.org/10.1172/jci.insight.169736DS1), show that TRAF4 deficiency significantly reduced the percentage and number of ST2^+^FOXP3^+^ cells, while having no effect on ST2^–^FOXP3^+^ cells. This suggests that the IL-33/TRAF4 axis is critical for the development of ST2^+^ Tregs but not ST2^–^ Tregs. Although ST2^+^ Th1 and ST2^+^ Th17 have also been reported in different experimental/physiological conditions (34, 35), we were unable to generate ST2^+^ Th1 or Th17 cells through in vitro polarization from naive CD4^+^ T cells. Further investigation is needed to determine the role of IL-33/TRAF4 in these 2 ST2^+^ Th subsets. To determine whether TRAF4-deficient Th2 cells have a global impairment in activation, we conducted TCR ligation experiments using CD3/CD28 on mTh2 cells. As shown (Supplemental Figure 3, A–C), both TRAF4-deficient and TRAF4-sufficient mTh2 cells exhibited similar proliferation rates and Th2 cytokine production upon TCR ligation. This suggests that TRAF4 deficiency does not globally impair mTh2 activation.

### T cell–intrinsic TRAF4 is critical for the expansion of ST2^+^ mTh2 cells in IL-33–induced airway inflammation.

**Figure 2:**
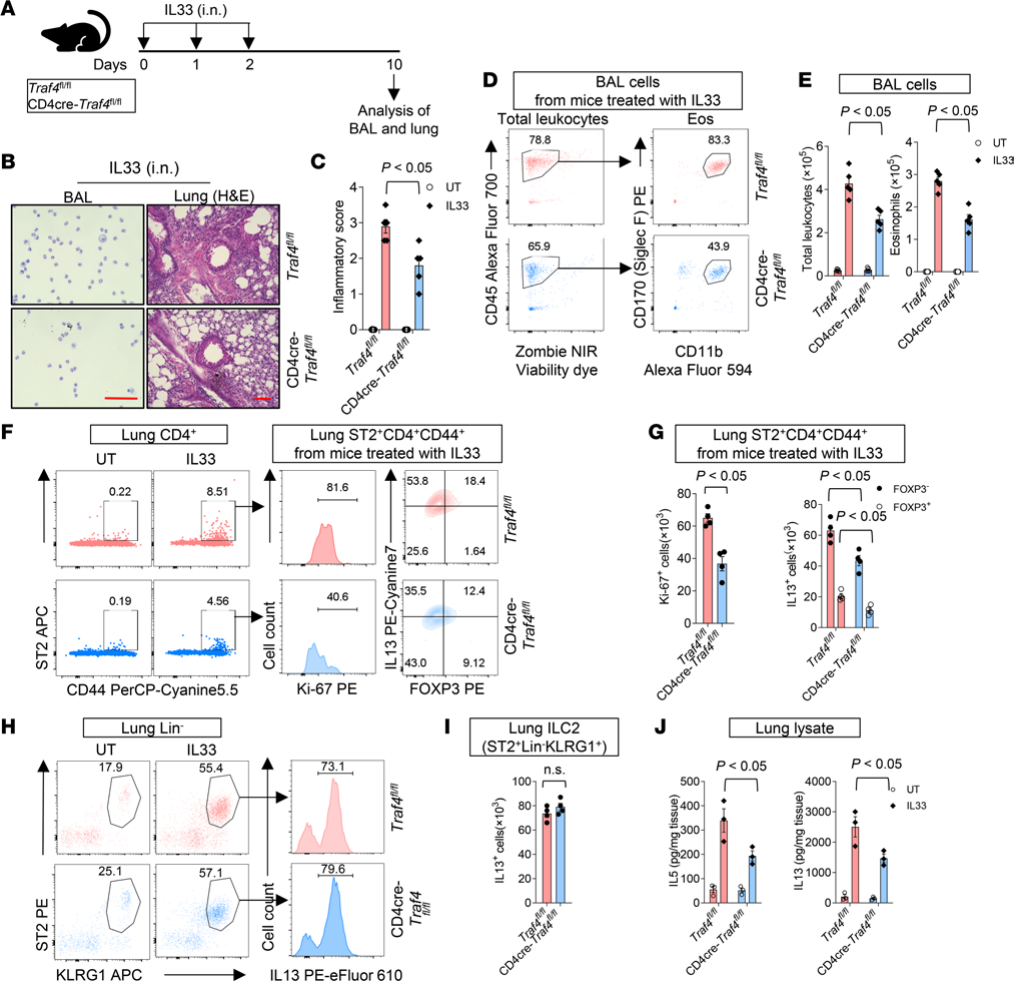
T cell–specific TRAF4 deficiency alleviates IL-33–induced type 2 airway inflammation. (**A**) Eight-week-old female T cell–specific TRAF4-deficient (CD4-cre *Traf4^fl/fl^*) and TRAF4-sufficient (*Traf4^fl/fl^*) mice were treated as indicated in the experimental protocol. (**B** and **C**) Giemsa staining of BAL cells and H&E staining of lung tissue sections. Scale bars (red), 100 μm. BAL, bronchoalveolar lavage. (**C**) Inflammation scores of tissue sections. (**D**) Flow cytometry plot of eosinophils (CD45^+^CD11b^+^SiglecF^+^) in the BAL. Eso, eosinophils. (**E**) Total leukocytes (CD45^+^) and eosinophils (CD45^+^CD11b^+^SiglecF^+^) in the BAL. (**F**) Flow cytometry analysis of lung CD4^+^ cells. (**G**) Absolute numbers of lung Ki-67^+^ST2^+^CD4^+^CD44^+^ and IL-13^+^ST2^+^CD4^+^CD44^+^ cells. (**H**) Flow cytometry analysis of lung Lin^–^ (CD45^+^CD3^–^LY6G^–^LY6C^–^CD11b^–^B220^–^TER-119^–^) cells. (**I**) Absolute numbers of lung Lin^–^ST2^+^KLRG1^+^ cells. (**J**) IL-5 and IL-13 protein concentrations in lung tissue were quantified by ELISA. Plotted data were shown as means ± SEM. Statistical analysis was performed with 1-way ANOVA (**G** and **I**) or 2-way ANOVA (**C**, **G**, and **J**). All data are representative of 3 independent experiments.

We next investigated the effect of T cell–specific TRAF4 deficiency on the proliferation of ST2^+^ mTh2 cells in vivo. We performed an IL-33 intranasal injection experiment on T cell–specific TRAF4-deficient (CD4-cre *Traf4^fl/fl^*) and TRAF4-sufficient control mice (*Traf4^fl/fl^*) (Figure 2A). As indicated by lung histology (Figure 2, B and C) and BAL counts — total leukocytes (CD45^+^) and eosinophils (CD45^+^CD11b^+^SiglecF^+^) (Figure 2, D and E) — TRAF4-deficient mice displayed reduced pulmonary inflammation as compared with TRAF4-sufficient mice. The basal numbers of ST2^+^CD44^+^CD4^+^ (mTh2) cells in lung tissue (<1% of total lung CD4^+^ cells) were low in both CD4-cre *Traf4^fl/fl^* and *Traf4^fl/fl^* mice (Figure 2F). IL-33 induced greater expansion of ST2^+^CD44^+^CD4^+^ cells (8.5% of lung CD4^+^ cells) in *Traf4^fl/fl^* mice than that (4.6% of lung CD4^+^ cells) in CD4-cre *Traf4^fl/fl^* mice (Figure 2, F and G). Consistently, ST2^+^CD44^+^CD4^+^ cells from *Traf4^fl/fl^* mice had more proliferating potential (as indicated by the staining of cell proliferating marker Ki-67) than those from CD4-cre *Traf4^fl/fl^* mice (Figure 2, F and G). Since it has been known that IL-33 also induces the proliferation of ST2^+^ Treg (FOXP3^+^) cells (31, 32, 36, 37), we then further gated ST2^+^CD44^+^CD4^+^ cells into FOXP3^+^ (Treg) and FOXP3^–^ (mTh2) cells. Interestingly, both FOXP3^+^ and FOXP3^–^ cells were found to produce type 2 cytokine (as indicated by IL-13 intracellular staining), and IL-33–induced numbers of IL-13^+^FOXP3^+^ and IL-13^+^FOXP3^–^ cells were higher in *Traf4^fl/fl^* mice than those in CD4-cre *Traf4^fl/fl^* mice (Figure 2G). We also examined IL-33–induced expansion of ILC2s (ST2^+^Lin^–^ KLRG1^+^), another major producer of type 2 cytokines in lung tissue exposed to IL-33 (38). In both IL-33–treated TRAF4-sufficient and TRAF4-deficient mice, we observed comparable numbers of IL-13^+^ ILC2s (Lin-ST2^+^KLRG1^+^) and Ki-67^+^ ILC2s (Figure 2, H and I), implying that the reduced expansion of lung IL-13–producing ST2^+^CD44^+^CD4^+^ mTh2 cells in TRAF4-deficient (CD4-cre *Traf4^fl/fl^*) mice may be the cause of the attenuated eosinophilic airway inflammation. Consistently, IL-33–induced production of type 2 cytokines (IL-5 and IL-13) was also significantly reduced in lung tissue in TRAF4-deficient (CD4-cre *Traf4^fl/fl^*) mice as compared with control (*Traf4^fl/fl^*) mice (Figure 2J). IL-33 has been shown to induce the expression of its own receptor ST2 (IL1RL1) (39). The ST2 protein has 2 major isoforms: a soluble circulating form (sST2) and a membrane-bound receptor form (ST2). Studies have shown that patients with asthma exhibit elevated levels of sST2 protein in their blood. This protein acts as a negative feedback regulator of IL-33 signaling, which helps control airway inflammation (40, 41). We examined the sST2 protein levels in the BAL and plasma of TRAF4-deficient and TRAF4-sufficient mice following IL-33 injection. While naive mice from both genotypes had comparable BAL and plasma sST2 levels, CD4-cre *Traf4^fl/fl^* mice had significantly lower sST2 in both BAL and plasma compared with *Traf4^fl/fl^* mice following IL-33 injection (Supplemental Figure 4, A and B). This suggests that TRAF4 is critical for IL-33–induced sST2 protein in vivo. Taken together, T cell–specific TRAF4 deficiency impairs the expansion of IL-13–producing ST2^+^CD4^+^CD44^+^ mTh2 cells in the lung tissue and eosinophilic pulmonary inflammation in the IL-33 injection model.

### T cell–specific TRAF4 deficiency attenuates Alternaria-induced type 2 airway inflammation.

**Figure 3:**
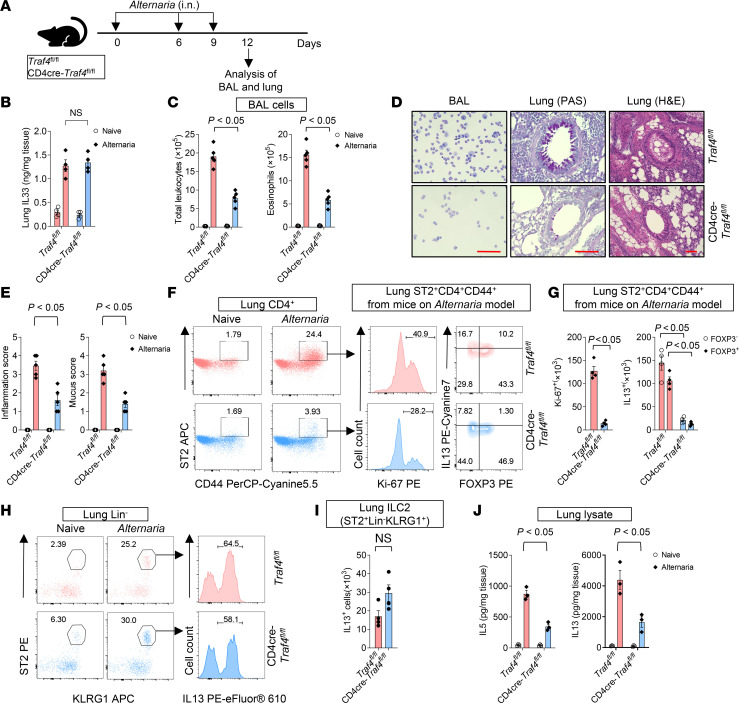
T cell–specific TRAF4 deficiency mitigates *Alternaria*-induced type 2 airway inflammation. (**A**) Ten-week-old female T cell–specific TRAF4-deficient (CD4-cre *Traf4^fl/fl^*) and TRAF4-sufficient (*Traf4^fl/fl^*) mice were treated as indicated in experimental protocol. (**B**) Lung IL-33 protein concentrations were measured by ELISA. (**C**) Total leukocytes (CD45^+^) and eosinophils (CD45^+^CD11b^+^SiglecF^+^) in the BAL. (**D**) Giemsa staining of BAL cells and H&E staining of lung tissue sections. Scale bars (red), 100 μm. (**E**) Inflammation scores and mucus scores of tissue sections. (**F**) Flow cytometry analysis of lung CD4^+^ cells. (**G**) Absolute numbers of lung Ki-67^+^ST2^+^CD4^+^CD44^+^ and IL-13^+^ST2^+^CD4^+^CD44^+^ cells. (**H**) Flow cytometry analysis of lung Lin^–^ cells. (**I**) Absolute numbers of lung Lin^–^ST2^+^KLRG1^+^ILC2s. (**J**) Lung IL-5 and IL-13 protein levels were measured by ELISA. Plotted data were shown as means ± SEM. Statistical analysis was performed with 1-way ANOVA (**G** and **I**) or 2-way ANOVA (**B**, **C**, **E**, **G**, and **J**). All data are representative of 3 independent experiments.

We then sought to investigate the impact of T cell–specific TRAF4 deficiency on allergen-induced pulmonary inflammation. The fungal allergen (*Alternaria alternate*) is a rapid IL-33 inducer in the airways and is associated with asthma severity and exacerbations (42). We thus used an *Alternaria*-driven type 2 asthma model in which IL-33 expression was substantially increased in lung tissue (Figure 3, A and B). *Alternaria* induced the expression of IL-33 at comparable levels in both *Traf4^fl/fl^* and CD4-cre *Traf4^fl/fl^* mice (Figure 3B). However, in comparison with TRAF4-sufficient control (*Traf4^fl/fl^*) mice, TRAF4-deficient (CD4-cre *Traf4^fl/fl^*) mice displayed significantly reduced eosinophilic inflammation, as indicated by total leukocytes (CD45^+^) and eosinophils (CD45^+^CD11b^+^SiglecF^+^) in the BAL (Figure 3C) and lung histology (H&E and periodic acid–Schiff [PAS] staining) (Figure 3, D and E). Previous studies have reported increased levels of IL-17A and neutrophils in mice challenged with *Alternaria* (43). In our *Alternaria* model (Figure 3A), we detected low levels of IL-17A (<40 pg/mL) and small percentages of neutrophils (<2%) in the BAL in the challenged mice (Supplemental Figure 5, A and B). Compared with TRAF4-sufficient mice, TRAF4-deficient mice had reduced levels of IL-17A and neutrophils in the BAL following *Alternaria* challenge. The Th17/IL-17A axis is known to be associated with neutrophilic airway diseases (44–46). TRAF4 is an essential signaling molecule in the TGF-β pathway (47), which is important for Th17 differentiation and development. Therefore, TRAF4 deficiency leads to reduced levels of IL-17A (the major cytokine of Th17) and neutrophils. *Alternaria*-induced proliferation (as indicated by Ki-67 staining) of ST2^+^CD4^+^CD44^+^ cells as well as expansion of IL-13^+^ST2^+^CD4^+^CD44^+^ cells were significantly reduced in CD4-cre *Traf4^fl/fl^* mice as compared with control *Traf4^fl/fl^* mice (Figure 3, F and G). Following *Alternaria* challenge, the numbers of IL-13–producing mTh2 (FOXP3^–^ST2^+^CD4^+^CD44^+^) and Treg (FOXP3^+^ST2^+^CD4^+^CD44^+^) cells in lung tissue were also significantly lower in CD4-cre *Traf4^fl/fl^* mice than those in *Traf4^fl/fl^* mice (Figure 3G). In contrast, the number of IL-13^+^ ILC2s (ST2^+^Lin^–^KLRG1^+^) was similar in both CD4-cre *Traf4^fl/fl^* and *Traf4^fl/fl^* mice (Figure 3, H and J). Additionally, we observed that TRAF4-deficient lungs when exposed to *Alternaria* produced fewer type 2 cytokines (IL-5 and IL-13) than control lungs, which was in line with the changes in pulmonary pathology (Figure 3J). It should be noted that lung IL-13^+^ST2^+^CD4^+^CD44^+^ mTh2 cells outnumbered IL-13^+^ ILC2s by more than 5 times in TRAF4-sufficient *Traf4^fl/fl^* mice subjected to the *Alternaria* model (Figure 3, G and I), indicating that IL-13^+^ST2^+^CD4^+^CD44^+^ mTh2 cells contribute more to *Alternaria*-induced airway inflammation than IL-13^+^ ILC2s. This is in contrast with the almost equivalent numbers of IL-13^+^ST2^+^CD4^+^CD44^+^ mTh2 cells and IL-13^+^ ILC2s in the lung tissue from the *Traf4^fl/fl^* mice subjected to IL-33 injection (Figure 2, G and I). As a result, the mice in the *Alternaria* model exhibited a more dramatic phenotype caused by T cell–specific TRAF4 deficiency than the mice in the IL-33 injection model (Figure 2, B–E, and Figure 3, C–E). The above results indicate that T cell–specific TRAF4 is crucial for allergen-induced eosinophilic airway inflammation and expansion of IL-13^+^ST2^+^CD4^+^CD44^+^ mTh2 cells in the lung.

### The intrinsic TRAF4 is crucial for IL-33–induced expansion of OVA_323-339_–specific ST2^+^ mTh2 cells and development of eosinophilic airway inflammation in adoptive transfer mice.

**Figure 4:**
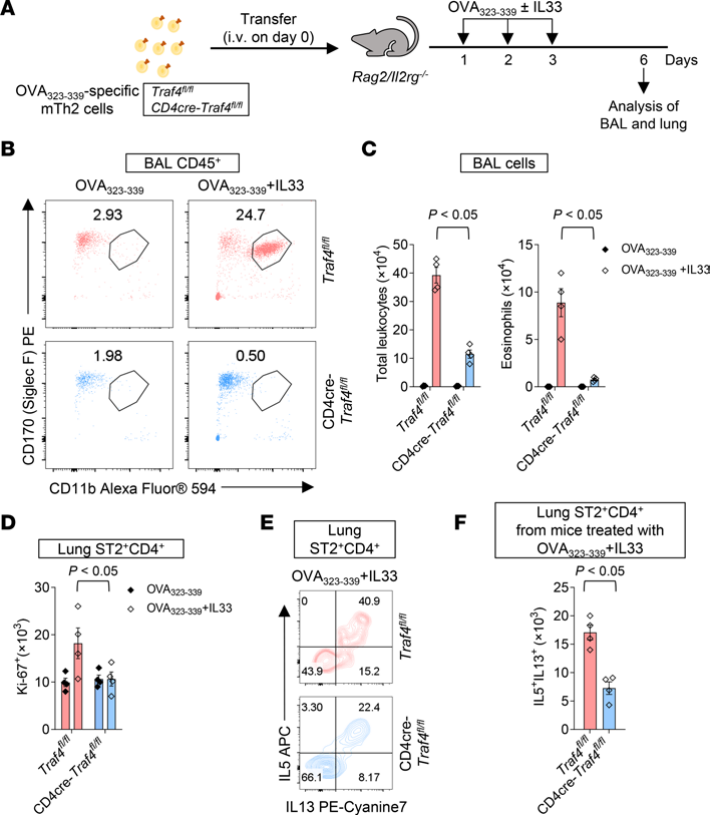
The intrinsic TRAF4 is crucial for IL-33–induced expansion of OVA_323-339_–specific ST2^+^ mTh2 cells and development of eosinophilic airway inflammation in adoptive transfer mice. (**A**) Ten-week-old female *Rag2* *Il2rg^–/–^* mice were adoptively transferred with T cell–specific TRAF4-deficient (CD4-cre *Traf4^fl/fl^*) and TRAF4-sufficient (*Traf4^fl/fl^*) mTh2 cells and challenged with OVA peptide with or without IL-33 as indicated in the experimental protocol. (**B**) Flow cytometry plot of BAL eosinophils (CD45^+^CD11b^+^SiglecF^+^). (**C**) Total leukocytes (CD45^+^) and eosinophils (CD45^+^CD11b^+^SiglecF^+^) in the BAL. (**D**) Absolute numbers of lung Ki-67^+^ST2^+^CD4^+^CD44^+^ cells. (**E**) Flow cytometry plot of lung IL-13^+^IL-5^+^ST2^+^CD4^+^ cells. (**F**) The absolute number of lung IL-13^+^IL-5^+^ST2^+^CD4^+^ cells. Plotted data were shown as means ± SEM. Statistical analysis was performed with 1-way ANOVA (**F**) or 2-way ANOVA (**C** and **D**). All data are representative of 3 independent experiments.

Since IL-33– and *Alternaria*-induced type 2 inflammation involves induction of both ST2^+^ mTh2 and ST2^+^ILC2 lymphocytes (Figure 2, H and I, and Figure 3, H and I), we sought an in vivo model in which the ST2^+^ mTh2 cells are the sole lymphocyte population driving type 2 airway inflammation. We performed the adoptive transfer experiment with *Rag2^–/–^IL2rg^–/–^* (R2G2) mice (genetically lacking ILCs, T cells, B cells, and NK cells) as recipients (Figure 4A). In this experimental model, R2G2 mice were subjected to adoptive transfer of OVA_323-339_–specific TRAF4-sufficient (*Traf4^fl/fl^*) or TRAF4-deficient (CD4-cre *Traf4^fl/fl^*) ST2^+^ mTh2 cells so that the impact of ST2^+^ mTh2 (not ST2^+^ ILC2s and ST2^+^ Tregs) was manifest. After transfer, the R2G2 recipient mice were challenged (i.n.) with a low dose (5 μg) of OVA_323-339_ peptide along with sham or IL-33 treatment. OVA_323-339_ peptide induced similar low-grade eosinophilic airway inflammation, as indicated by the numbers of eosinophils (CD45^+^CD11b^+^SiglecF^+^) in the BAL, of both TRAF4-sufficient and TRAF4-deficient mice (Figure 4, B and C). However, after being cotreated with IL-33, recipient mice transferred with TRAF4-sufficient (*Traf4^fl/fl^*) cells developed significantly more eosinophilic inflammation in the lung than the mice transferred with TRAF4-deficient (CD4-cre *Traf4^fl/fl^*) cells (Figure 4, B and C). Likewise, the numbers of proliferating ST2^+^CD4^+^ cells (Ki-67^+^) and cytokine-producing IL-5^+^IL-13^+^ST2^+^CD4^+^ cells in IL-33/OVA_323-339_–treated mice transferred with TRAF4-sufficient cells were greater than those in IL-33/OVA_323-339_–treated mice transferred with TRAF4-deficient cells (Figure 4, D–F). The data presented above suggest that the intrinsic TRAF4 deficiency impairs the IL-33–induced expansion of antigen-specific ST2^+^ mTh2 cells as well as the development of eosinophilic inflammation in the absence of ST2^+^ ILC2 and other lymphocytes in the lungs of adoptive transfer mice.

### TRAF4 is required for IL-33–mediated PI3K/AKT and ERK1/2 pathways as well as signature genes involved in T cell growth and proliferation in ST2^+^ mTh2 cells.

**Figure 5:**
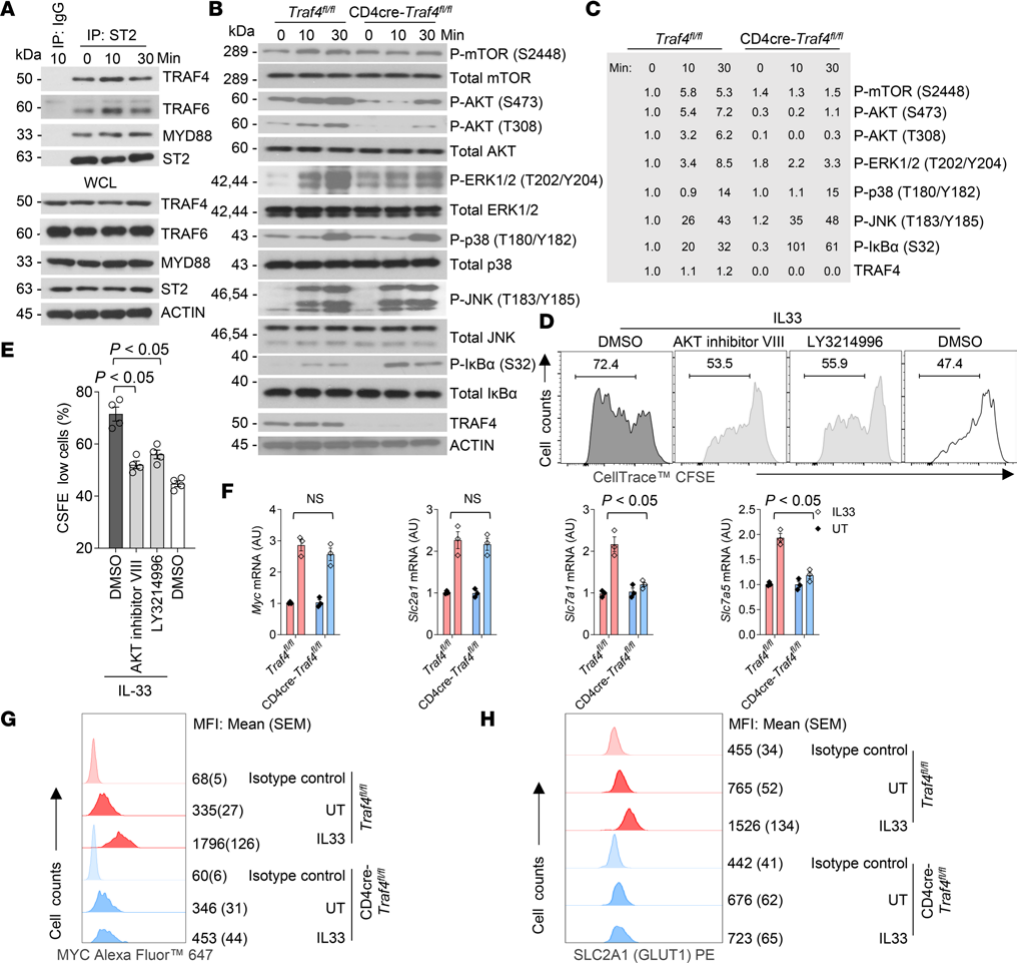
TRAF4 is required for IL-33–mediated AKT/mTOR and ERK1/2 pathways as well as signature genes involved in T cell growth and proliferation in ST2^+^ mTh2 cells. (**A**) Cell lysates of in vitro–polarized mouse mTh2 cells treated with or without IL-33 were subjected to the co-immunoprecipitation assay with anti-ST2 antibody, followed by Western blot analysis with the indicated antibodies. WCL, whole cell lysate. (**B**) In vitro–polarized mouse mTh2 cells (starved in cytokine-free medium for 24 hours) were treated with IL-33 at different time points, and cell lysates were analyzed by Western blot with indicated antibodies. (**C**) The density of each protein band was quantified by ImageJ (NIH, V. 1.51) and normalized to its total protein or Actin (for TRAF4). Then the fold induction was calculated related to the value of *Traf4^fl/fl^* sample at time 0 minutes (which was set to 1) for each protein. (**D**) Histograms of ST2^+^ mTh2 cells (treated with IL-33 along with sham or indicated pathway inhibitors) subjected to CFSE cell proliferation assay. (**E**) Frequency of CFSE^lo^ cells. (**F**) Real-time PCR analysis of the mRNA abundance in mTh2 cells treated with IL-33 for 24 hours. (**G** and **H**) Representative histogram blots showing the intracellular expression of MYC and the surface expression of GLUT1 on mTh2 cells treated with sham or IL-33 for 24 hours. Plotted data were shown as means ± SEM. Statistical analysis was performed with 1-way ANOVA (**E**) or 2-way ANOVA (**F**). All data are representative of 2 independent experiments.

We next investigated the molecular mechanism of TRAF4 in IL-33–induced ST2^+^ mTh2 proliferation. We performed a co-immunoprecipitation experiment with an antibody targeting endogenous IL-33 receptor (ST2) using in vitro–polarized ST2^+^ mTh2 cells. As shown in Figure 5A, upon IL-33 stimulation, TRAF4 was recruited to ST2 along with known signaling molecules MYD88 and TRAF6, indicating that TRAF4 forms a proximal receptor complex with them. In CD4-Cre–transgenic mice, the initiation of Cre expression occurs during the late double-negative stage of T cell development, leading to the deletion of loxP-flanked genes by the double-positive stage (48). As anticipated, we found that in CD4-cre *Traf4^fl/fl^* mice, TRAF4 expression was nearly completely eliminated in both CD4^+^ and CD8^+^ cells, while remaining unaffected in innate lymphoid cells (ILCs) (Supplemental Figure 8 and Figure 5B). It is known that IL-33 induces multiple pathways (e.g., NF-κB, MAPKs, PI3K/AKT/mTOR) in different types of cells (17). We examined the activation of these signaling pathways by IL-33 in ST2^+^ mTh2 cells cultured from TRAF4-sufficient and TRAF4-deficient mice (Figure 5, B and C). We found that IL-33–induced activation of AKT/mTOR and ERK1/2 (as shown by the phosphorylation of the indicated signaling molecules) was greatly reduced in TRAF4-deficient cells as compared with that in TRAF4-sufficient cells. TRAF4 deficiency also diminished IL-33–induced phosphorylation of ribosomal protein S6 (p-S6) and eukaryotic initiation factor 4E binding protein 1, key downstream effectors of mTOR for protein synthesis (Supplemental Figure 6, A and B). In contrast, TRAF4 deficiency did not affect the activation of JNK and p38 MAPKs (as shown by the phosphorylation of the indicated kinases) by IL-33, while the activation of NF-κB (as indicated by p-IκBα) was negatively regulated by TRAF4 deficiency. We then assessed the effect of AKT inhibitor VIII (an allosteric inhibitor of AKT1 and AKT2) (49, 50) and LY3214996 (a selective ERK1/2 inhibitor) (51) on IL-33–induced ST2^+^ mTh2 proliferation by a cell tracing experiment with CFSE. The results showed that IL-33–induced mTh2 proliferation and Th2 cytokines (IL-5 and IL-13) were significantly reduced by AKT inhibitor VIII and attenuated by LY3214996 (Figure 5, D and E, and Supplemental Figure 7, A and B). These findings suggest that TRAF4 regulates IL-33–induced mTh2 proliferation via the AKT/mTOR and ERK1/2 pathways. To further investigate the critical role of TRAF4 in the proliferation of mTh2 induced by IL-33, we compared the IL-33–induced expression of the signature genes involved in T cell growth and proliferation between TRAF4-sufficient and TRAF4-deficient ST2^+^ mTh2 cells (Figure 5F). *Myc* is the master transcription factor required for TCR-induced T cell growth and proliferation (52, 53), while *Scl2a1* (glucose transporter protein type 1, GLUT1), *Scl7a1* (cationic amino acid transporter 1, CAT1), and *Scl7a5* (L-type amino acid transporter 1, LAT1) are important nutrient transporters that are dependent on *Myc* to support T cell growth and proliferation. We found that the transcripts of *Myc*, *Scl2a1*, *Scl7a1*, and *Scl7a5* were noticeably upregulated by IL-33 stimulation in TRAF4-sufficient (*Traf4^fl/fl^*) ST2^+^ mTh2 cells. However, TRAF4 deficiency did not affect the mRNA expression of *Myc* and *Scl2a1* but impaired transcription of *Scl7a1* and *Scl7a5* in ST2^+^ mTh2 after IL-33 stimulation for 24 hours (Figure 5F). Interestingly, TRAF4 deficiency reduced the intracellular protein level of *Myc* and the surface protein expression of *Scl2a1* upregulated by IL-33 (Figure 5, G and H). The above results suggest that a subset of IL-33–induced genes involved in cell growth and proliferation are TRAF4-dependent in ST2^+^ mTh2 cells.

## Discussion

Asthma is a prevalent immunologic disorder, around half of which is driven by type 2 inflammation and characterized by increased eosinophilic infiltration in the lung. Although the majority of type 2 asthmatics are responsive to steroid-based therapy, a subgroup of patients, especially those with persistent eosinophilic inflammation, are refractory to steroid treatment and require high-dose steroids to control the symptoms (54). Increased IL-33 expression is associated with severe and refractory asthma (6, 7). IL-33 administration to the mouse airway induces the expansion and cytokine production of both ST2^+^ mTh2 cells and ST2^+^ ILC2s, contributing to antigen-dependent and -independent type 2 airway inflammation (55). Steroid-resistant (SR) subsets of ST2^+^ mTh2 cells and ST2^+^ ILC2s have been reported in allergic human patients or mice in experimental models of eosinophilic airway diseases (9, 56–58). Targeting IL-33–induced ST2^+^ mTh2 cells and ST2^+^ ILC2s, the major producers of type 2 cytokines in the airway, may be effective for SR eosinophilic airway inflammation. Interestingly, 2 recent phase II clinical trials have indicated that mAbs blocking either IL-33 or its receptor ST2 were beneficial for certain subsets of patients with asthma (15, 16). The current study identified TRAF4 as a crucial regulator of the proliferation of ST2^+^ mTh2 cells induced by IL-33 in in vitro cell culture studies as well as in in vivo murine models of type 2 airway inflammation, which may open up avenues for the development of new therapeutic strategies for SR eosinophilic asthma.

TRAF4 has been reported as a positive regulator for cell growth/proliferation pathways (AKT, ERK1/2, and ERK5) induced by EGF, TGF-β, and IL-17A signaling, contributing to the proliferation and migration of cancer cells (47, 59–61). AKT/mTOR and ERK1/2 pathways are also critical for CD4^+^ T cell proliferation in response to TCR activation and IL-2 (62). In the current study, we found that TRAF4, along with MYD88, is recruited to the IL-33 receptor (ST2) upon IL-33 stimulation, forming a proximal signaling complex in ST2^+^ mTh2 cells. Furthermore, TRAF4 deficiency impaired IL-33–induced activation of AKT/mTOR and ERK1/2 pathways as well as the proliferation of ST2^+^ mTh2 cells. TRAF4 is known to bind phosphatidylinositol phosphate (PIP) lipids (e.g., PIP2 and PIP3) on cell membranes (63), which may facilitate PI3K kinase recruitment and the activation of downstream AKT/mTOR pathway. Conversely, we also discovered that TRAF4 negatively regulates p-IκB, an important upstream event promoting NF-κB translocation into the nucleus and activation. Activated IL-33 receptor recruits IRAKs, which bind and activate TRAF6 (a well-known upstream signaling molecule for the NF-κB pathway) through TRAF-binding sites (64). There likely exists a competition between TRAF4 and TRAF6 for TRAF-binding sites on IRAKs in the IL-33 pathway. A similar mechanism has been depicted in the IL-17A pathway in which TRAF4 and TRAF6 compete for the TRAF-binding motifs on Act1 (the essential signaling adaptor for IL-17A receptor) (65).

Prior research has highlighted the innate (TCR-independent) immune functions of ST2^+^ mTh2 cells. In vitro–polarized ST2^+^ mTh2 cells can produce IL-5 and IL-13 upon IL-33 stimulation alone or with a STAT5 activator (e.g., IL-2, IL-7, or TSLP) (39). In vivo studies also revealed that ST2^+^ mTh2 cells in lung tissue produced type 2 cytokines in response to IL-33 or IL-33–inducing allergens in a TCR-independent manner (66). In the current study, we also observed that IL-33 was able to induce the production of IL-5 and IL-13 in our in vitro–polarized ST2^+^ mTh2 cells in the presence of IL-2 and IL-7. Moreover, the administration of IL-33 and IL-33–inducing allergen (e.g., *Alternaria*) to the airway was able to induce the expansion of IL-13^+^ST2^+^CD4^+^ mTh2 cells in lung tissue. Therefore, our studies provide additional evidence to support the innate functions of ST2^+^ mTh2 cells in antigen-independent type 2 airway inflammation.

ST2^+^ Tregs (characterized by FOXP3 expression) are an important subset of CD4^+^ T cells that are found at barrier sites (e.g., gut, lung). ST2^+^ Tregs exert either suppressive or proinflammatory functions in different experimental contexts. In respiratory infection models, IL-33 maintained FOXP3 expression and suppressive function of Tregs, which appeared inclined to acquire Th17 phenotype (upregulation of RORC and IL-17 expression) in the absence of ST2 (37). In a mouse model of T cell–induced colitis, ST2 expression by Tregs was critical to prevent the onset of disease in the gut. Conversely, the additional studies showed that ST2^+^ Tregs exhibited Th2-biased characteristics, producing type 2 cytokines (IL-5 and IL-13) and even losing their ability to suppress effector T cells when stimulated in vitro with IL-33 (31, 67). Airway administration of IL-33 to mice impaired established immunologic tolerance to the OVA antigen, which was accompanied by an increased number of ST2^+^ Tregs expressing IL-5 and IL-13 (31). Interestingly, in an HDM-induced type 2 pulmonary inflammation model, ST2^+^ Tregs activated by IL-33 were able to suppress IL-17–producing γ/δ T cells but not adaptive Th2 immune response (68). The findings from the current study demonstrated that airway administration of IL-33 or IL-33–inducing allergen (*Alternaria*) promoted the expansion of IL-13–expressing FOXP3^–^ (mTh2) and FOXP3^+^ (Treg) ST2^+^CD4^+^ cells in the lung, suggesting that both mTh2 cells and Tregs may contribute to airway inflammation as the producers of type 2 cytokines. Additionally, we found that TRAF4 deficiency impaired IL-33–induced propagation of both FOXP3^–^ mTh2 cells and FOXP3^+^ Tregs. However, the precise role of TRAF4 in Tregs needs to be further investigated using FOXP3-specific TRAF4-deficient mice in type 2 airway inflammation.

The transcription factor MYC is considerably increased after TCR ligation, and this promotes the transcription of vital enzymes and transporters involved in a variety of metabolic pathways during the activation and growth of CD4^+^ T cells. (52, 53). MYC deficiency drastically decreases T cell growth and proliferative capacity (52, 53, 69). Multiple mechanisms, including transcriptional, posttranscriptional, and posttranslational controls, are involved in the regulation of MYC expression (70–77). In particular, MYC protein level is regulated by phosphorylation/dephosphorylation-mediated stabilization and degradation events (74, 75, 77). ERK activity stabilizes MYC by phosphorylation at serine 62, whereas glycogen synthase kinase 3β destabilizes it by phosphorylation at threonine 58, which can be blocked by the PI3K/AKT pathway (69, 74, 78). In the present study, we observed that TRAF4 deficiency had little impact on MYC transcription but markedly reduced MYC protein level in IL-33–stimulated ST2^+^ mTh2 cells, suggesting that TRAF4 may affect the posttranslational control of MYC protein through ERK1/2 and PI3K/AKT pathways.

MYC-mediated induction of amino acid transporters is pivotal for the bioenergetic and biosynthetic pathways in TCR-activated T cells (52). In the present study, IL-33–induced transcription of 2 MYC-dependent amino acid transporters (*Scl7a1* and *Slc7a5*) was impaired in TRAF4-deficient ST2^+^ mTh2 cells. *Slc7a1* encodes CAT1, which is a key transporter for l-arginine and l-ornithine. Arginases convert l-arginine to l-ornithine, which is an important precursor for the synthesis of polyamines (spermidine, spermine, and putrescine). Polyamine metabolites have been associated with cell growth and proliferation (79). Arginase 1 deficiency in ILC2s leads to reduced polyamine synthesis and impaired cell proliferation (80). Likewise, the reduced surface expression of *Scl7a1* in TRAF4-deficient ST2^+^ mTh2 cells may impair the uptake of arginine, leading to reduced polyamine synthesis and cell proliferation. *Scl7a5* encodes the light chain unit of the large neutral amino acid (LNAA) transporter 1 (LAT1), which is the main transporter for LNAAs like leucine and methionine. Leucine is an important nutrient signal for mTORC1 activation (81), and methionine (the starting amino acid for each protein) availability sets the limit for the overall efficiency of protein synthesis. *Slc7a5^–/–^* T cells have defective mTOR activation and proliferation upon TCR ligation, which recapitulates the phenotype of *Myc^–/–^* T cells (52, 82). The current study indicates that IL-33, similar to TCR ligation, induces MYC-dependent expression of amino acid transporters to meet the increased metabolic needs for the growth and proliferation of ST2^+^ mTh2 cells. It will be interesting to map the complete proteomes of ST2^+^ mTh2 cells reprogrammed by IL-33 stimulation and understand the effect of TRAF4 deficiency on the overall amino acid transport.

The present study found that surface expression of glucose transporter GLUT1 was greatly attenuated by TRAF4 deficiency in IL-33–treated ST2^+^ mTh2 cells, though the transcript abundance of GLUT1 was not affected. Despite MYC being previously reported to be critical for mRNA expression of GLUT1 of T cells upon TCR activation (53), more recent studies indicate that GLUT1 transcription is MYC independent (52). Interestingly, intracellular trafficking of GLUT1 has been shown to depend on the PI3K/AKT pathway in different cell types (83–85). Therefore, the impaired AKT pathway in TRAF4-deficient ST2^+^ mTh2 cells may impede the trafficking of GLUT1, resulting in the tapered surface expression of GLUT1. Future research should interrogate the specifics of how TRAF4 deficiency impacts IL-33–induced glucose metabolic pathways in ST2^+^ mTh2 cells.

Although the current study has revealed an essential role of TRAF4 in ST2^+^ mTh2 cells in the contexts of IL-33–mediated allergen-independent and -dependent type 2 airway inflammation, several questions remain to be addressed. For example, what is the impact of TRAF4 RING domain E3 activity on IL-33–mediated pathways and functions in ST2^+^ mTh2 cells? It would also be interesting to know whether TRAF4 contributes to the SR characteristics of ST2^+^ mTh2 cells given its critical role in promoting cell growth and proliferation. Finally, a comprehensive analysis of IL-33–induced transcriptomes and proteomes altered by TRAF4 deficiency is required to achieve an in-depth understanding of TRAF4’s functions in ST2^+^ mTh2 cells.

## Methods

### Mice.

B6. OT-II TCR-transgenic mice (stock no: 004194) and CD4-Cre–transgenic mice (stock no: 022071) were purchased from The Jackson Laboratory. *Rag2^–/–^*/*Il2rg*^–/–^ mice (Model 4111-F) were obtained from Taconic Biosciences. *Traf4^fl/fl^* mice were generated by Cyagen Biosciences using gene-targeting technology. The experimental mice were female and age matched (8–12 weeks). All mice were bred on C57BL/6 background and maintained under specific pathogen–free conditions. All animal experiments were performed with the approval of the Cleveland Clinic’s IACUC.

### Reagents and cell culture.

Mouse IL-2 (catalog 575402), mouse IL-4 (catalog 547302), mouse IL-33 (catalog 580502), anti-mouse CD3ε (145-2C11), anti-mouse CD28 (37.51), human TGF-β1 (catalog 781802), anti-mouse IL-4 mAb (clone 11B11), anti-mouse IFN-γ mAb (XMG1.2), and Apotracker Green were purchased from BioLegend. Antibodies for TRAF4 (D1N3A), MyD88 (D80F5), p-mTOR (S2448, D9E), mTOR (7C10), p-AKT (S473, D9E), p-AKT (T308, D25E6), AKT (11E7), p-JNK (T183/Y185, 81E11), SAPK/JNK (no. 9252), p-p38 (T180/Y182, D3F9), p38 (D13E1), p-ERK1/2 (T202/Y204, D13.14.4E), Erk1/2 (137F5), p-IκBα (S32, 14D4), IκBα (L35A5), TRAF6 (D21G3), and β-Actin (8H10D10) were obtained from Cell Signaling Technology. Mouse T1/ST2 antibody (DJ8) and rabbit polyclonal against ST2 (ab228543) were obtained from MD Bioproducts and Abcam, respectively. AKT inhibitor VIII (CAS 612847-09-3) and LY3214996 (CAS 1951483-29-6) were purchased from Cayman Chemical. Primary mouse CD4^+^ T cells were cultured in complete TexMACS Medium (Miltenyi Biotec, 130-097-196) supplemented with 10% FBS, 50 μM β-mercaptoethanol, 100 U/mL penicillin, and 100 μg/mL streptomycin. CD8^+^ cells and ILCs were isolated from spleen using MojoSort Mouse CD8 T Cell Isolation Kit and Biotin anti-mouse Lineage Panel from BioLegend.

### In vitro polarization of ST2^+^ mTh2 cells.

Naive CD4^+^ T cells (CD4^+^CD44^–^CD62L^+^) were isolated from mouse spleen by negative selection using the Naive CD4^+^ T Cell Isolation Kit (Miltenyi Biotec, 130-104-453). Purified naive T cells were activated with plate-bound anti-CD3ε (5 μg/mL) and soluble anti-CD28 (2 μg/mL) and cultured in complete TexMACS Medium under Th2-polarizing conditions (50 ng/mL IL-4, 10 ng/mL IL-2, 10 μg/mL anti–IFN-γ) for 5 days to get mTh2 cells. Then cells were washed with PBS and maintained in the medium with IL-2 and IL-7 (10 ng/mL, respectively) for 10 days to generate ST2^+^ mTh2 cells.

### In vitro polarization of ST2^+^ Tregs.

Naive CD4^+^ T cells were isolated as described above. The cells were then activated with CD3/CD28 and cultured in complete TexMACS Medium under Treg-polarizing conditions (10 ng/mL IL-2, 10 ng/mL human TGF-β1, 10 μg/mL anti–IL-4, 10 μg/mL anti–IFN-γ) for 5 days in the presence of 10 ng/mL IL-33.

### IL-33–induced type 2 airway inflammation.

Recombinant mouse IL-33 (1 μg/mouse) was i.n. administered in 40 μL sterile saline for 3 consecutive days. On day 10, BAL and lung tissue were harvested and analyzed.

### Alternaria-induced type 2 airway inflammation.

*Alternaria alternata* (M1, Stallergenes Greer, 50 μg/mouse) dissolved in 40 μL sterile saline was administered (i.n.) on days 0, 6, and 9. BAL and lungs were harvested and analyzed on day 12.

### ST2^+^ mTh2 adoptive transfer model.

We bred TRAF4-sufficient *Traf4^fl/fl^* and TRAF4-deficient CD4-*Traf4^fl/fl^* mice onto OT-II TCR-transgenic mice to obtain *Traf4^fl/fl^* OT-II TCR and CD4-*Traf4^fl/fl^* OTII TCR-transgenic mice. OVA_323–339_–specific ST2^+^ mTh2 cells were generated from naive CD4^+^ T cells isolated from *Traf4^fl/fl^* OTII TCR and CD4-*Traf4^fl/fl^* OTII TCR as described above. Then these cells were transferred (i.v.) into 10-week female R2G2 mice (10 × 10^6^ cells per mouse) and challenged (i.n.) with OVA_323–339_ (5 μg/mL) peptide with or without IL-33 (1 μg/mouse) for 3 consecutive days (days 0–2 after transfer). BAL cell counting and tissue collection were performed 3 days after the last antigen challenge.

### Real-time quantitative PCR.

Total RNA was isolated using Quick-RNA Microprep Kit (ZYMO Research, catalog R1050) as per the manufacturer’s instructions. First-strand cDNA was synthesized by ZymoScript RT PreMix Kit (ZYMO Research, catalog R3012). All gene expression results were expressed as an AU relative to the abundance of *Gapdh* mRNA. The primers used for the real-time PCR are *Myc* (forward: TCGCTGCTGTCCTCCGAGTCC; backward: GGTTTGCCTCTTCTCCACAGAC), *Slc2a1* (forward: GCTTCTCCAACTGGACCTCAAAC; backward: ACGAGGAGCACCGTGAAGATGA), *Slc7a1* (forward: CTCCTGGCTTACTCTTTGGTGG; backward: GATCTAGCTCCTCGGTGGTTCT), *Slc7a5* (forward: GGTCTCTGTTCACGTCCTCAAG; backward: GAACACCAGTGATGGCACAGGT), and *Gapdh* (forward: CATCACTGCCACCCAGAAGACTG; backward: ATGCCAGTGAGCTTCCCGTTCAG).

### Histology.

Lung tissue was fixed in 10% neutral-buffered formalin. The fixed tissue was then dehydrated, embedded in paraffin, and cut into 5 μm sections. These sections were deparaffinized and stained with H&E and PAS (MilliporeSigma, catalog 395B-1KT). At least 20 bronchioles (>200 μm) were examined for each slide; 10 fields were counted for each slide. The inflammation score was determined based on the infiltration of cells, using a 5-point system: 0 for no cells, 1 for a few cells, 2 for 1 ring of cells, 3 for 2–4 rings of cells, and 4 for more than 4 rings of cells. The mucus score was calculated as the ratio of PAS-positive cells to total epithelial cells, with a score of 0 indicating no positive cells, 1 indicating less than 25% positive cells, 2 indicating 25%–50% positive cells, 3 indicating 50%–75% positive cells, and 4 indicating more than 75% positive cells.

### Differential BAL cell counting.

BAL cell counts were determined by cytospin slide preparation followed by Wright-Giemsa staining with Hema 3 Manual Staining System and Stat Pack (Thermo Fisher Scientific, catalog 22-122911)

### Flow cytometry and antibodies.

Lung tissues were perfused by PBS and cut into small pieces before subjection to the digestion of Liberase (MilliporeSigma, catalog 5401119001) plus DNase I (MilliporeSigma, catalog 11284932001) for 45 minutes at 37°C. The single-cell solution was obtained using a cell strainer (40 μm) (Thermo Fisher Scientific, catalog 08-771-1), followed by treatment with RBC lysis buffer (BioLegend, catalog 420301). BAL cells and single lung cells were first gated by (FSC-A × SSC-A) to remove debris, followed by (FSC-A × FSC-H) to remove doublets, and then the dead cells were excluded using Zombie NIR Fixable Viability dye (BioLegend, catalog 423105) (Supplemental Figure 1, A and B). The gated live cells were blocked by anti–mouse CD16/32 Ab (clone 2.4G, BD Biosciences, catalog 553142) to avoid nonspecific binding to Fc receptors before surface or intracellular staining. Flow cytometry antibodies are from BioLegend — Alexa Fluor 700 anti-mouse CD45 (30-F11), APC anti-mouse Ly-6G (1A8), PerCP/Cyanine5.5 anti-mouse CD44 (IM7), FITC anti-mouse Lineage Cocktail (anti-mouse CD3e, 145-2C11; anti-mouse Ly-6G/Ly-6C, RB6-8C5; anti-mouse CD11b, M1/70; anti-mouse CD45R/B220, RA3-6B2; anti-mouse TER-119/Erythroid cells, Ter-119), PE anti-mouse FOXP3 (150D), PE anti-mouse Ki-67 (16A8) — BD Biosciences — PE anti-mouse CD170 (SiglecF, E50-2440), PE-CF594 anti-mouse CD11b (M1/70), APC anti-mouse KLRG1 (MAFA) — Cell Signaling Technology — Myc (D84C12) mAb — and Thermo Fisher Scientific — PE-Cyanine7 anti-mouse IL13 (eBio13A); PE-eFluor 610 anti-mouse IL13 (eBio13A), FITC anti-P-AKT1(S473) (AktS473-C7), PE Anti-P-4EBP (V3NTY24), APC Anti-P-S6 (cupk43k), PE-cy7 Anti-P-mTOR (MRRBY). Myc mAb (D84C12) was labeled by Alexa Fluor 647 Antibody Labeling Kit (Invitrogen, catalog A20186). Surface staining was conducted using Cell Staining Buffer (BioLegend, catalog 420201). Intracellular and nuclear staining was carried out with eBioscience Intracellular Fixation & Permeabilization Buffer (catalog 88-8823-88) and eBioscience Foxp3/Transcription Factor Staining Buffer Set (catalog 00-5523-00), respectively. The sample analysis was performed using BD Biosciences LSRFortessa Cell Analyzer.

### Cell proliferation assay.

Cells were washed with PBS twice and labeled with CellTrace CFSE (Thermo Fisher Scientific, catalog C34554) (1 μM in PBS, 20 minutes, room temperature). Then the cells were incubated for 5 minutes with 5 volumes of complete TexMACS medium to remove any free dye before they were pelleted and suspended in fresh complete TexMACS medium for an additional 10 minutes and treated as indicated. The analysis was completed using BD LSRFortessa Cell Analyzer with FITC and Alexa Fluor 488 channel (488 nm excitation and a 530/30 nm bandpass emission filter).

### ELISA.

Supernatants were collected from cell cultures, and protein extracts of lung tissue were obtained using T-PER Tissue Protein Extraction Reagent (Thermo Fisher Scientific, catalog 78510). The levels of mouse IL-17A, IL-5, and IL-13 were measured with DuoSet ELISA kits (R&D Systems, DY421, DY405, and DY413, respectively). Mouse IL-33 was quantified by ELISA Kit (Thermo Fisher Scientific, catalog 88-7333-22). Soluble ST2 was measured by ST2 (IL-33R) Mouse Uncoated ELISA Kit with Plates (Thermo Fisher Scientific, catalog 88-9334-22).

### Co-immunoprecipitation.

Cells were lysed on ice in Pierce IP Lysis Buffer (25 mM Tris-HCl pH 7.4, 150 mM NaCl, 1 mM EDTA, 1% NP-40, and 5% glycerol) (Thermo Fisher Scientific, catalog 87788) supplemented with cOmplete Protease Inhibitor Cocktail (MilliporeSigma, 11697498001), followed by centrifugation (10,000*g*, 10 minutes, at 4°C). The supernatant was immunoprecipitated using 2 μg/mL anti-mouse ST2 antibody and Pierce Protein G Magnetic Beads (Thermo Fisher Scientific, catalog 88848), followed by Western blot analysis.

### Statistics.

We used 1-way or 2-way ANOVA. *P* < 0.05 was indicated as significant. *P* > 0.05 was indicated as not significant. GraphPad Prism (V9.4.0) was used for all tests and calculations.

### Study approval.

All animal studies were approved by the Cleveland Clinic’s IACUC.

### Data availability.

To find the values for all data points in the graphs, please refer to the Supporting Data Values file.

## Author contributions

CL and XL conceived and designed all the experiments. JX, CL, XC, WL, KB, WQ, WML, and LH performed the experiments and collected/analyzed the data. CL wrote the manuscript. CL and XL supervised the whole project.

## Supplementary Material

Supplemental data

Supporting data values

## Version 1 08/22/2023

In-Press Preview

## Version 2 09/22/2023

Electronic publication
