# Supplementary material for: TRAF4 is crucial for ST2+ memory Th2 cell expansion in IL-33–driven airway inflammation
Source: JCI Insight. 2023 Sep 22;8(18):e169736. doi: 10.1172/jci.insight.169736 (PMC10561728; doi:10.1172/jci.insight.169736)
Supplement: Supplemental data [file jciinsight-8-169736-s063.pdf]

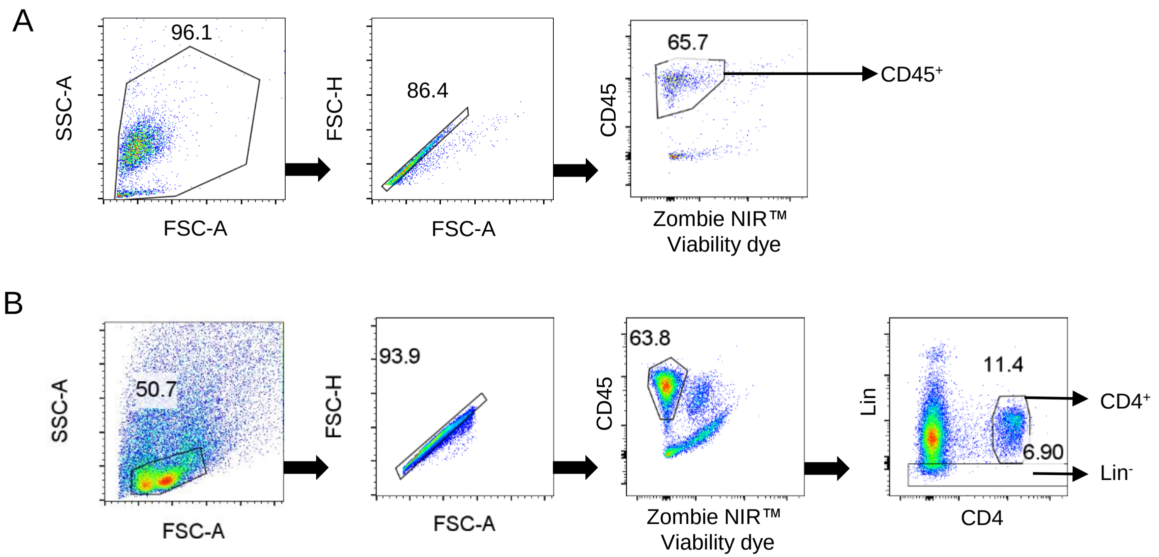

Supplemental Figure 1. Gating strategies for live CD45$^+$, CD4$^+$ and lineage negative (Lin$^-$) cells. Bronchoalveolar lavage **(**BAL) and single lung cells were first gated by (FSC-A × SSC-A) to remove debris, followed by (FSC-A × FSC-H) to remove doublets, and then the dead cells were excluded using Zombie NIR^{TM} Fixable Viability dye. (A) Gating CD45$^+$cells. (B) Gating CD4$^+$ and Lin$^-$ cells.

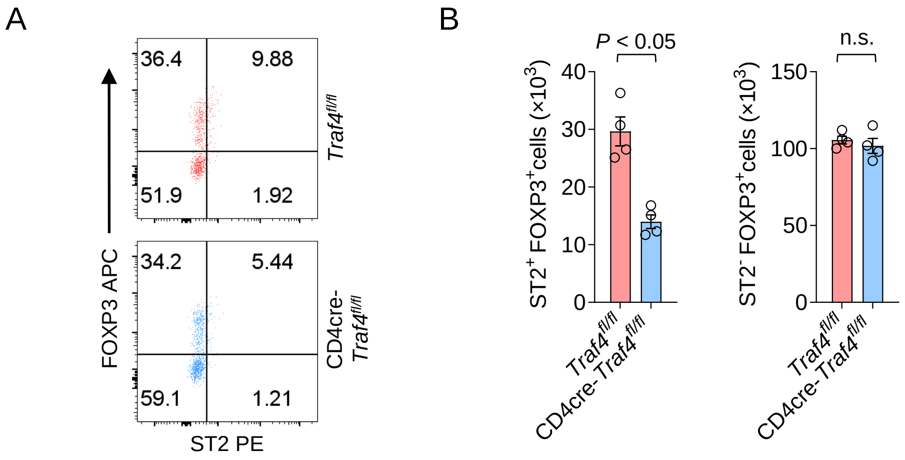

Supplemental Figure 2. TRAF4 is critical for IL-33-mediated induction of ST2⁺Treg cells in vitro. (A) Naive CD4 T cells isolated from TRAF4-deficient (CD4cre-*Traf4*$^{fl/fl}$) and TRAF4-sufficient (*Traf4*$^{fl/fl}$) mice were cultured under Treg condition (see Methods) in the presence of IL-33 for 5 days. (B) Absolute numbers of $ST2^{+}FOXP3^{+}$ and $ST2^{-}FOXP3^{+}$ Treg cells. Data are presented as means ± SEM and were analyzed using one-way ANOVA. These results are representative of two independent experiments.

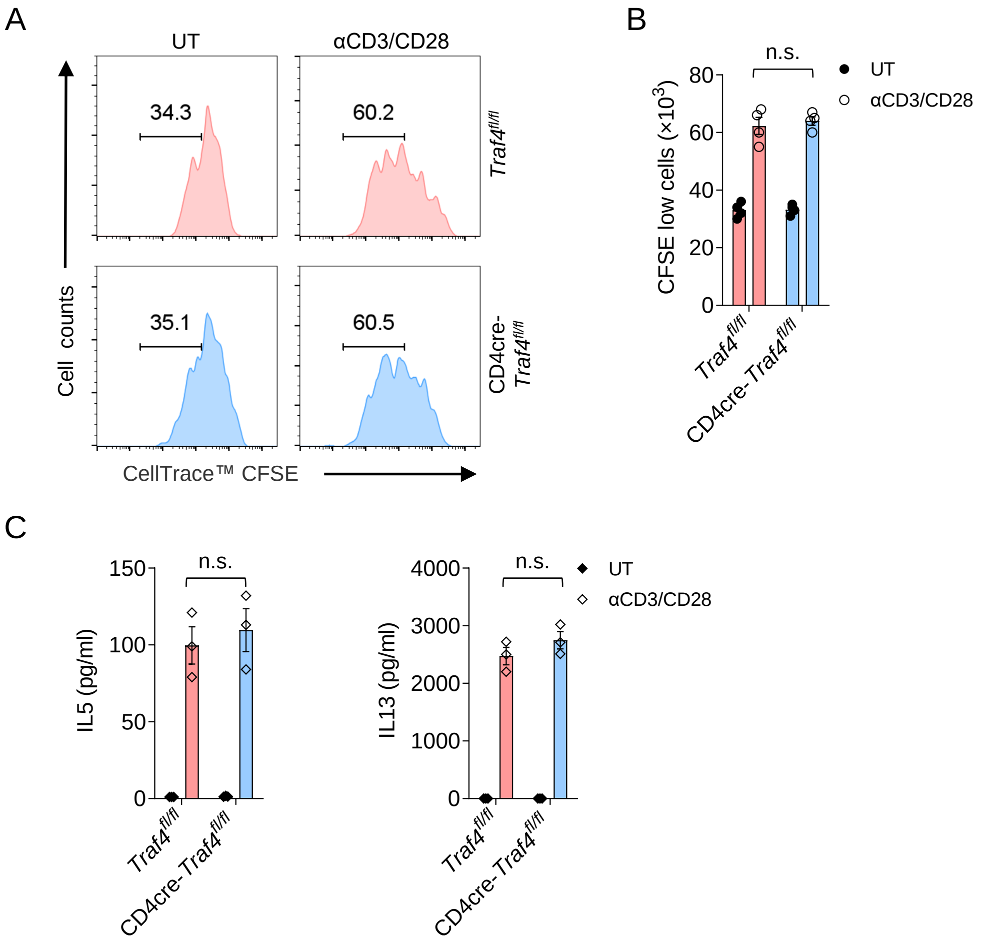

Supplemental Figure 3. TRAF4 is not required for TCR-induced proliferation of memory Th2 cells (mTh2). (A) Histograms of αCD3/CD28-treated (3 d) ST2$^+$ mTh2 cells subjected to CFSE cell proliferation assay. (B) Absolute numbers of CFSE low cells. (C) IL-5 and IL-13 protein concentrations in cell medium were quantified by ELISA. Data are presented as means ± SEM and were analyzed using two-way ANOVA. These results are representative of two independent experiments.

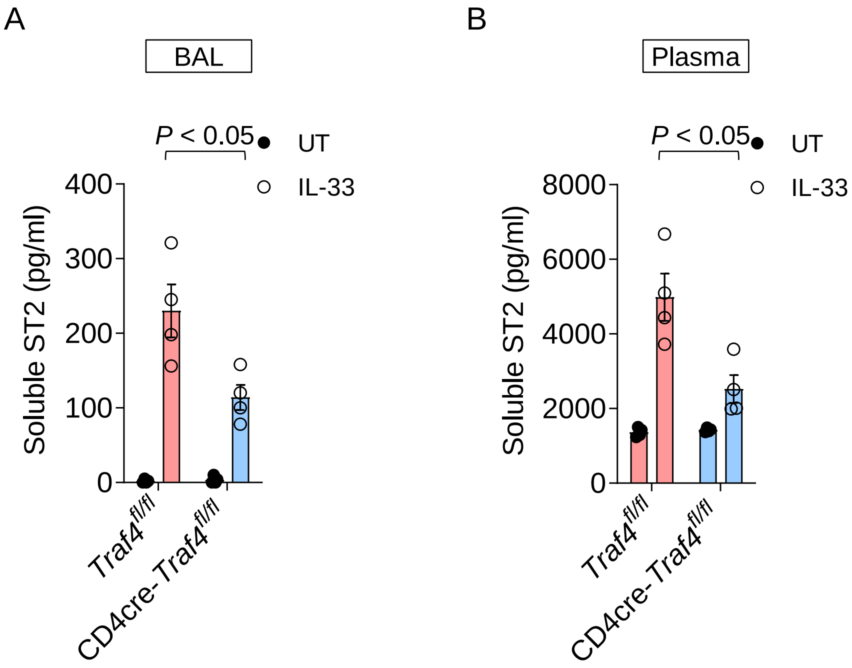

Supplemental Figure 4. TRAF4 deficiency impairs the levels of soluble ST2 induced by IL-33 in both bronchoalveolar lavage (BAL) fluid and plasma. TRAF4-deficient (CD4cre-$Traf4^{fl/fl}$) and TRAF4-sufficient ($Traf4^{fl/fl}$) mice were administrated intranasal IL-33 injections (as described in Figure 2A). The levels of soluble ST2 (sST2) in the BAL and plasma were subsequently measured by ELISA. Data are presented as means ± SEM and were analyzed using two-way ANOVA. These results are representative of two independent experiments.

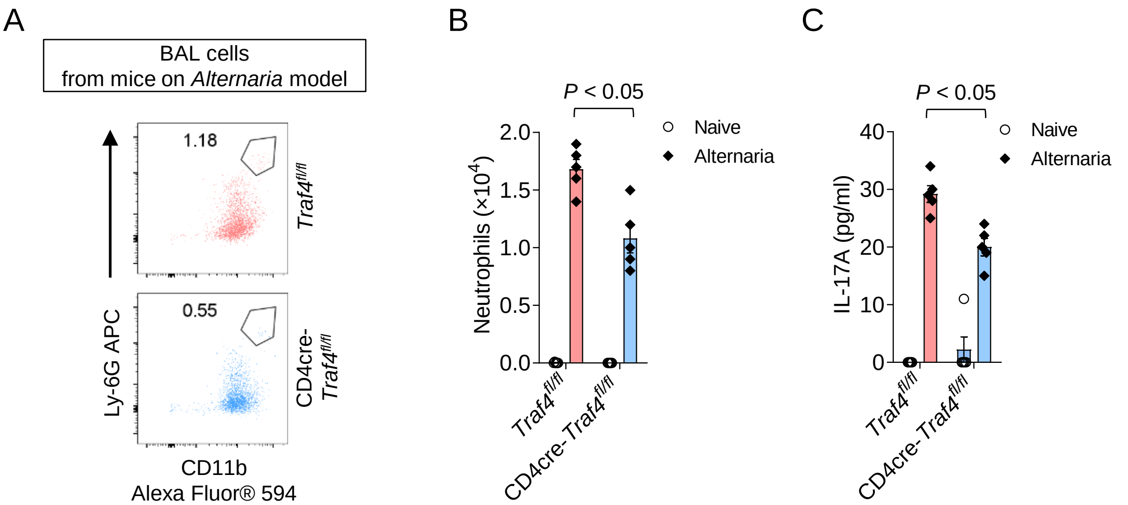

Supplemental Figure 5. TRAF4 is critical for the induction of airway neutrophils and IL-17A by *Alternaria* challenge. TRAF4-deficient (CD4cre-*Traf4*$^{fl/fl}$) and TRAF4-sufficient (*Traf4*$^{fl/fl}$) mice were subjected to *Alternaria* model as described in Figure 3A. (A) Flow cytometry analysis of neutrophils ($CD45^+CD11b^+Ly6\text{-}G^+$) in the bronchoalveolar lavage (BAL). (B) Absolute numbers of neutrophils in the BAL. (C) IL-17A protein level in the BAL. Data are shown as means ± SEM and were analyzed by two-way ANOVA. The results are representative of two independent experiments.

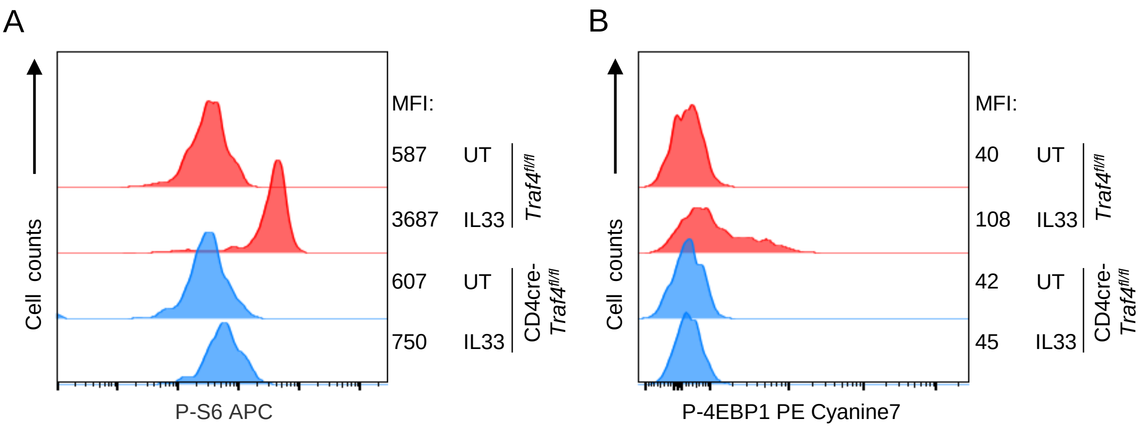

Supplemental Figure 6. TRAF4 deficiency diminishes IL-33-induced phosphorylation of S6 ribosomal protein and 4EBP1 in mTh2 cells. Representative histogram blots showing the surface expression of phospho-S6 ribosomal protein (P-S6) and phospho-4EBP1 (P-4EBP1) on TRAF4-deficient (CD4cre-*Traf4*$^{fl/fl}$) and TRAF4-sufficient (*Traf4*$^{fl/fl}$) mTh2 cells treated with sham or IL-33 for 24 h. MFI, Mean fluorescence intensity. Plotted data were shown as means ± SEM. All data are representative of two independent experiments.

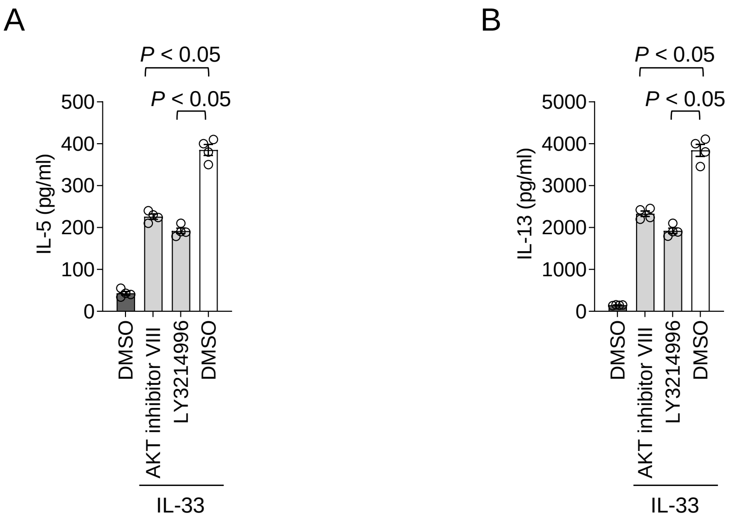

Supplemental Figure 7. Inhibition of AKT and ERK pathways reduces IL-33-induced type 2 cytokine production. mTh2 cells were treated with sham or IL-33 along with indicated pathway inhibitors for 24 h. IL-5 (A) and IL-13 (B) protein concentrations in the cell medium were quantified by ELISA. Plotted data were shown as means ± SEM. Statistical analysis was performed with one-way ANOVA followed by Turkey's multiple comparison test. All data are representative of three independent experiments.

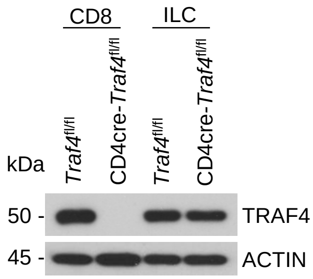

Supplemental Figure 8. TRAF4 expression in CD8 cells and innate lymphocytes (ILCs). CD8 cells and ILCs were isolated from TRAF4-deficient (CD4cre-*Traf4*$^{fl/fl}$) and TRAF4-sufficient (*Traf4*$^{fl/fl}$) mice. Cell lysates were analyzed by western blot with indicated antibodies.
